# Representation in genetic studies affects inference about genetic architecture

**DOI:** 10.64898/2026.01.12.699135

**Published:** 2026-01-13

**Authors:** Jared M. Cole, Shane Rybacki, Samuel Pattillio Smith, Olivia S. Smith, Arbel Harpak

**Affiliations:** 1 Department of Integrative Biology, University of Texas at Austin, Austin, TX, USA; 2 Department of Population Health, University of Texas at Austin, Austin, TX, USA

## Abstract

Knowledge of a trait’s “genetic architecture,” namely the joint distribution of allele frequencies of causal variants and the direction and magnitude of their effects, is essential to understanding its evolution and underlying biology. Inferences about genetic architecture are based on data collected in heterogeneous ways in cohorts recruited through heterogeneous mechanisms. This heterogeneity, in turn, results in differences in genotype, environment and trait distributions across cohorts. For instance, the UK Biobank (UKB) aimed for broad population representation, whereas FinnGen drew heavily from clinical registries enriched for diagnosed health conditions. Here, we asked whether representation in genetic studies influences inferences about genetic architectures. Using GWAS data from the UKB, FinnGen, and All of Us (AoU), we find that some summaries of a trait’s genetic architecture, such as effective polygenicity, vary little across biobanks. Others, like SNP heritability, are systematically lower in one biobank (AoU) than in another (UKB) across almost all traits examined, even when matching samples such that they have similar genetic ancestry compositions. This result aligns with other recent evidence that biobanks with disease-enriched recruitment have lower heritability than population-based biobanks, which may consequently reduce power to detect genotype–phenotype associations. We highlight a third case, where a summary of genetic architecture varies considerably but not systematically across traits and biobanks. Such is the case for the mean direction of allelic effects (“sign bias”). For example, 72% of rare minor alleles affecting type 2 diabetes risk are inferred to be risk-increasing based on AoU data, while nearly 100% are inferred to be risk-increasing based on UKB data. We hypothesize that the inferred sign bias is heavily influenced by the skewness of the trait distribution in the study and is otherwise independent of other study or trait characteristics, including whether the trait is binary or quantitative. We provide strong support for this hypothesis through simulations and data from the three biobanks: the variation in inferred sign bias across traits and biobanks is explained remarkably well (81% and 97% of variance explained for trait-associated and for a random set of SNPs, respectively) solely by the trait’s skewness in the biobank, with residual biobank-specificity explaining little (incremental adjusted $R^{2}=2.4\%$). These results illustrate that inferences about the map between genetic and phenotypic variation can depend on representation in genetic studies in surprising ways.

## Introduction

Characterizing the mapping of genetic variation onto trait variation is central to understanding the biological mechanisms underlying complex traits and their evolution. Summaries of this mapping for a given trait are often referred to jointly as its “genetic architecture.” More formally, the genetic architecture of a trait comprises the joint distribution of frequencies and effects (including both magnitudes and directions) of causal variants^1,2^. Genome-wide association studies (GWAS) have allowed researchers to estimate variant-trait associations for hundreds of complex traits and diseases and thus characterize several aspects of their underlying genetic architectures^3^.

Through this effort, we have learned that many traits are highly polygenic, i.e. most trait variation is explained by small effects of numerous alleles^1,4–7^. Allelic effects typically span multiple orders of magnitude (consisting of a few large effects amid many tiny ones^8,9^, and variants often exhibit pleiotropy, influencing multiple traits simultaneously^10,11^. Evolutionary forces such as mutation, natural selection, and genetic drift together mold genetic architecture, producing characteristic relationships between effect magnitude and allele frequency. One famous example is the negative monotonic relationship between the average magnitude of allelic effects and their frequency^1,12,13^.

Since inferences about genetic architecture are key to our understanding of the evolution and biology of complex traits, and because they are often assumed to be intrinsic to traits, it is concerning when aspects of architecture are found to vary across studies^14–17^. This variation is often attributed primarily to variation in genetic ancestry across study cohorts, leading to variable genetic interactions^18–21^. However, studies also differ by contexts, such as country, healthcare systems, or recruitment strategies. As one example, a biobank that primarily recruits from specialty clinics will over-represent individuals with severe or multiple diseases (e.g., FinnGen), whereas a population-volunteer cohort (e.g., UK Biobank) will under-represent people who are socioeconomically disadvantaged or in poor health. Differences in cohort recruitment can influence cohort-level estimates of allelic effects themselves and subsequent summaries such as genetic correlations^22–24^. There are existing approaches that statistically adjust for heterogeneity across studies (e.g., inverse-probability weighting^25,26^, but those do not directly inquire about its drivers. Thus, it remains unclear how study cohort characteristics influence our inferences about genetic architecture.

Here, we ask how representation in genetic studies affects inferences about genetic architecture. We study differences in summaries of genetic architecture across three major biobanks with distinct emphases in study recruitment: a population-volunteer cohort (UK Biobank^27^), a cohort emphasizing inclusion of under-represented groups (All of Us^28^), and a diagnosis-enriched cohort (FinnGen^29^). We compare summaries across 11 traits spanning both quantitative traits and disease endpoints. We then focus on the mean direction of allelic effects (“sign bias”) as a tractable case study. Sign bias estimates vary considerably across biobanks for some traits. We hypothesize that trait skewness may affect virtually any method used to estimate sign bias, and show support for this hypothesis both with empirical analysis across biobanks and traits and in simulation studies under a null scenario of no true sign bias. Together, these findings show that our estimates of key summaries of genetic architecture can depend heavily on study recruitment and participation.

## Results

### Headline summaries of genetic architecture vary across biobanks.

We investigated whether inferences about genetic architectures could depend on characteristics of recruitment and participation in genetic studies. To do so, we compared summaries of genetic architecture as estimated using GWAS data from distinct biobanks.

In particular, we analyzed GWAS summary statistics on ancestry-matched samples (see Methods) for 11 traits from the UK Biobank (UKB^27^) and the All of Us (AoU^28^) databases, which include three morphological, three immunological, and four disease traits (Table S1). All traits were analyzed in their raw units, with no standardization or other transformation (see our reasoning for this choice in the Discussion). Using bivariate LD Score Regression (*LDSC*^30^), we estimated SNP heritability (SNP $h^{2}$) for each trait-biobank pair and calculated their cross-biobank genetic correlations. We also estimated the effective polygenicity of each trait, Effective polygenicity is a measure of how evenly the heritable signal is distributed across variants. If the majority of the trait’s heritability is due to only a few loci, then the effective polygenicity is small, but if it is due to many loci across the genome, the effective polygenicity is large^31,32^. We note that this measure differs from commonly used measures of polygenicity that probe the number of variants with non-zero effects^32–35^.

**Figure 1:**
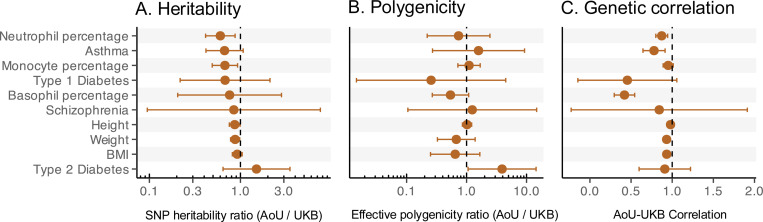
Summaries of genetic architecture vary across biobanks. For each trait (row), shown is the correspondence of estimates derived from GWAS performed in the All of Us (AoU) and UK Biobank (UKB) datasets. (A) The ratio of the SNP heritability estimated using LD Score Regression (with liability scale estimates for binary traits; observed scale estimates shown in Fig. S10). Across traits, AoU estimates of SNP heritability are systematically lower than AoU. (B) Ratio of effective polygenicity, which summarizes how diffuse vs. concentrated a trait’s heritability is across genomic loci. (C) Each row shows the genetic correlation, estimated using bivariate LD Score Regression, between allelic effects (on the same trait) estimated in UKB and AoU GWAS. Points represent estimates and horizontal bars represent 95% confidence intervals. Dashed vertical lines correspond to a ratio of 1 in A and B and a perfect genetic correlation $\left(r_{g}=1\right)$ in C. Alzheimer’s disease (AD) is omitted from the above panels because the SNP heritability estimates in both UKB and AoU were negative, precluding valid estimation of polygenicity and genetic correlations.

While the subsets of UKB and AoU we used for GWAS were, by design, similar in their genetic ancestry composition, summaries of genetic architecture often differed. In particular, with the exception of type 2 diabetes, SNP heritability estimates in AoU were lower than those in UKB (13.8% ± 3% lower on average across traits, estimated using Deming regression; Fig. 1A; Table S2). These results are consistent with prior reports of lower SNP heritability in AoU than UKB, even when GWAS in the two studies showed high genetic correlations^36^.

While heritability is a major focus in studies of genetic architecture, it is not an immutable property of a trait and should not be expected to be the same in distinct samples, even with close ancestry matching^22,37–39^. Here, the GWAS samples we analyzed are from different countries and societies. Further, they came about through fundamentally different recruitment strategies and different methods of data collection. These features of a study carry several implications for heritability. For example, the composition of (largely unmeasured) environmental and social factors is plausibly different across samples, resulting in differences in the extent of trait variation that is attributable to genetic variation.

Nevertheless, given the that statistical power to detect genotype-phenotype associations in biomedical GWAS directly depends on heritability^40–42^, it is noteworthy that heritability is systematically lower in AoU for the biomedical traits we examined. These results add to recent observations^16,36^ that together support a surprising conclusion: across 4 biobanks, it appears that SNP heritability tends to be lower in biobanks with disease-enriched recruitment. To our knowledge, our analysis is the first to demonstrate this systematic difference based on an ancestry-matched comparison. In the Discussion, we suggest a few hypotheses for the causes for this systematic difference.

Other quantities, like the dispersion of signal across variants (polygenicity) or the correlation of causal allelic effects (genetic correlation) among the samples are thought to be closer to trait-level properties because they do not directly depend on environmental variance and because causal genetic effects are expected to be similar across populations^18,19,32,40,43–45^. For most traits, effective polygenicity indeed differedonly a little between biobanks (Fig. 1B; Table S3), with type 2 diabetes and basophil percentage (AoU/UKB ratio of 0.25 ± 0.47 and 0.53 ± 0.5 respectively) showing the largest (though statistically insignificant) differences. However, genetic correlations between the two studies were significantly different from 1 for 6 out of the 10 traits examined (Fig. 1C; Table S4). The most notable examples are basophil percentage (AoU-UKB genetic correlation of 0.42 ± 0.06) and asthma (AoU-UKB genetic correlation of 0.78 ± 0.07). To confirm that these deviations from a genetic correlation of 1 are not a result of sampling noise, we estimated genetic correlations for random subsets of the UKB. Across traits, these never significantly deviated from one (Text S1; Table S4; Fig. S9). This finding is consistent with recent work showing that estimates of genetic correlations can depend on study design. Song et al.^23^ demonstrated that heritable participation biases, for example, can impact estimates of cross-trait genetic covariance.

### Sign bias: A case study of drivers of cross-biobank differences in genetic architecture.

The comparisons of SNP heritability, polygenicity and causal effects (probed through genetic correlations) may together suggest that genetic architectures vary across biobanks. To investigate the idea that this is largely a result of characteristics of the study sample, we examined a specific, tractable summary of allelic effects on complex traits, sign bias.

We define sign bias for a given set of variants and traits as the mean direction (sign) of allelic effects on the trait among these variants. The direction ought to be defined with respect to some well defined reference allele across variants. Here, we use the sign of the minor allele throughout. Hypothetically, sign bias can reflect typical biological mechanisms linking molecular phenotypes like gene expression levels and effects on traits^46–48^ or the mode of natural selection acting on complex traits^1^. For example, phenotypes under directional selection favoring an increase in population mean will accumulate more trait-decreasing minor alleles than trait-increasing ones, while those under stabilizing selection will show no sign bias^13,49–52^. At the same time, sign bias should be more tractable and less noisily estimated than the full distribution of allelic effects^53^.

In the Methods section, we describe a procedure that we developed to estimate sign bias for a set of variants. In short, we applied adaptive shrinkage (*ash*^54^), an empirical Bayes method that incorporates uncertainty in allelic effects, to sets of variants accounting for linkage disequilibrium. We estimated sign bias in sets of the most strongly associated SNPs across traits, bins of minor allele frequency and biobanks (Table S5). In what follows, we further focus on the most significantly associated SNPs for each trait (in Figs. S3, S5, and S7, we present results obtained with SNPs sampled randomly with respect to their trait association). Finally, to expand our scope, we also analyzed data from a third biobank, FinnGen^29^, for five corresponding traits also present in UKB and AoU for which the necessary summary data were publicly available. We note that for these FinnGen traits, the GWAS was performed by other researchers and did not include any ancestry matching to the UKB and AoU subsets that we analyzed.

**Figure 2:**
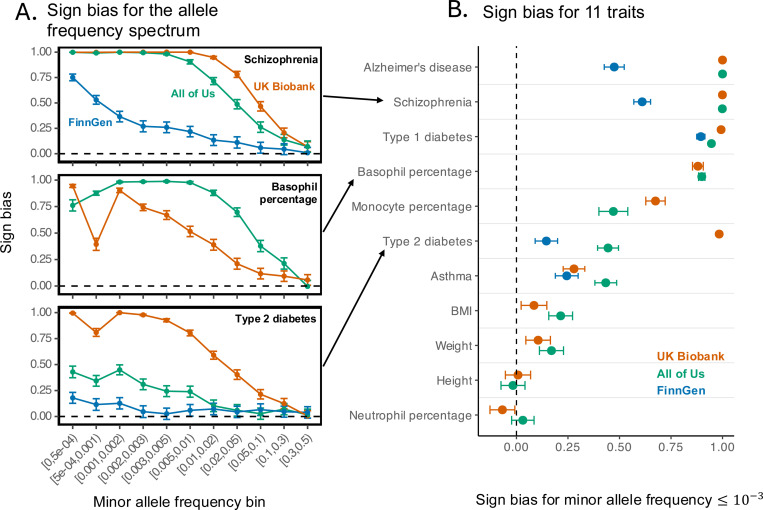
Sign bias varies across biobanks. We investigated the mean sign of allelic effects (“sign bias”) as an example summary of genetic architecture that can depend on the study cohort in which it is estimated. For each trait and a range of minor allele frequencies, we considered the sign of one SNP per approximately independent LD block. Here, we specifically used SNPs that were the most significantly associated with the trait in the block (Figs. S3, S5 show a reproduction of the analysis with randomly selected SNPs, i.e. regardless of the significance of trait association). The sign bias for each set of SNPs ranges from −1 (all SNPs are trait/risk decreasing) to 1 (all SNPs are trait/risk increasing). The sign bias is estimated using empirical Bayes adaptive shrinkage (*ash*) to account for measurement uncertainty. Points show point estimates and error bars show the 95% confidence intervals. Dashed horizontal and vertical lines show no sign bias (i.e., a value of 0). (A) Rare alleles are more sign-biased. Shown are three representative traits, with other traits shown in Fig. S2. (B) The estimated sign bias can vary substantially across three biobanks. Focusing on the rarer alleles (minor allele frequency of up to 0.1%), diseases tended to show the strongest positive sign bias, whereas morphometric traits (height, weight, BMI) showed little bias. Estimates derived from All of Us, the UK Biobank and FinnGen showed discrepancies, most notably for type 2 diabetes, Alzheimer’s disease, schizophrenia, and monocyte percentage.

Across traits, sign bias was negligible at common alleles (minor allele frequencies ≥ 0.1) but increased for rarer alleles (Figs. 2A, S2). The bias was most pronounced for binary disease traits, such as schizophrenia, type 1 diabetes, and Alzheimer’s disease (towards risk-increasing minor alleles) (Fig. 2B). In contrast, morphological traits (e.g., height, weight, BMI) showed little to no bias. Similar to other summaries of genetic architecture (Fig. 1), we found that sign bias estimates can differ substantially across biobanks. Across the UKB and AoU biobanks, type 2 diabetes (AoU/UKB ratio of 0.45 ± 0.03) and monocyte percentage (AoU/UKB ratio of 0.7 ± 0.06) are most discrepant. Across UKB and FinnGen as well as AoU and FinnGen, type 2 diabetes (FinnGen/UKB ratio of 0.15 ± 0.03; FinnGen/AoU ratio of 0.33 ± 0.07), Alzheimer’s disease (FinnGen/UKB and FinnGen/AoU ratios of 0.47 ± 0.02), and schizophrenia (FinnGen/UKB and FinnGen/AoU ratios of 0.61 ± 0.02) are the most discrepant.

That sign biases vary across biobanks indicates that they do not reliably reflect the impact of de-novo mutations on the trait. Similarly, sign biases are unlikely to reliably reflect the mode or intensity of natural selection, especially given that two of the three GWAS samples were ancestry-matched. What, then, could explain the marked variation in sign bias across biobanks?

### Skewness of the trait distribution in a study strongly determines sign bias.

We hypothesize that differences in estimated sign bias track differences in trait distribution across biobank cohorts, and specifically their skewnesses (standardized third central moments).

Our intuition stems from considering differences in discovery of risk vs. protective variants in binary disease traits. When cases are rare, observing even a few carriers of the same allele among cases hints at these alleles being risk-increasing. On the other hand, the identification of a protective effect of the same magnitude would require observing carriers of the allele who would have otherwise been likely cases (i.e., their background risk, excluding the focal variant, is high) but remain unaffected. Previous work showed that the strength of association between allele and disease status, and resultant test statistics, are indeed bounded by a function of the marginal frequency of the allele and the incidence of the disease^55–57^. When the minor allele increases risk for a rare disease, carriers can be concentrated among the relatively few cases, allowing a large (positive) correlation; yet if the minor allele is protective with the same magnitude of effect, its frequency among non-cases cannot be as high and its (negative) correlation with disease is constrained to be weaker.

The implications extend beyond the power asymmetry for large effect alleles, and extend to polygenic analyses of weaker allelic effects. In particular, consider a null of true risk-increasing and true risk-decreasing variants segregating at equal frequencies in a set of variants considered. There will be higher uncertainty of measurement of risk-decreasing effects, leading to larger standard errors, and higher rates of sign misclassification, resulting in an overall positive sign bias.

This intuition also extends to quantitative traits. To see this, consider first summarizing the imbalance between cases and non-cases in a binary trait by the skewness: when cases are rare in the sample, the trait distribution is highly right-skewed. Minor alleles that increase trait value can be found predominantly in the right tail, generating a high correlation between the variant and the trait. A major allele that decreases trait values cannot be found predominantly in the right tail of the distribution (because there are few such individuals and many copies of the allele) and so the correlation between the variant and the trait cannot be as high as it can for trait-increasing minor alleles^57^. The same logic applies to quantitative traits: as skewness increases, trait-decreasing minor alleles tend to be estimated with greater uncertainty in both magnitude and sign.

**Figure 3:**
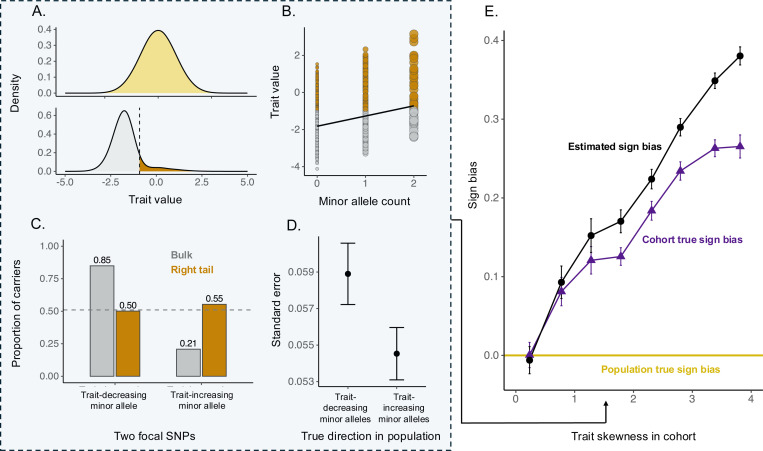
Simulation detailing how trait distribution skewness in a sample translates to inferred sign bias. We simulated a population of 2 × 10^6^ individuals with equal numbers of trait-increasing and trait-decreasing variants of identical magnitude and frequency. From this population, we drew a non-random sample of 10,000 individuals by preferentially sampling individuals from lower trait values, plus including a small uniformly random sample. Panels A-D show results for a sample with a skew of 1.5. We ran GWAS in this cohort and estimated the sign bias–the mean sign of effects of minor alleles. (A) Trait distributions. *Top*: population trait distribution (yellow); *Bottom*: trait distribution in a sampled GWAS cohort. The dashed line marks the 90th percentile of the cohort’s trait distribution and dark orange shading indicates the samples above it (the cohort “right tail” used in panels B–C). (B) Linear regression of the trait to one SNP with a trait-increasing minor allele in this cohort. Circle size is proportional to regression leverage; because the minor allele is rare, carriers (genotypes 1 and 2) have substantially higher leverage than non-carriers (genotype 0) and therefore have smaller regression residuals. Individuals above the 90th percentile of the sample’s trait distribution are shown in orange (C) We consider a focal pair of SNPs added for visualization with trait-increasing vs trait-decreasing minor alleles with the same magnitude of effect, both segregating at frequency 0.3 in the population. For each of the two SNPs, shown are the proportions of minor allele carriers among cohort individuals above (orange) and below (gray) the 90th percentile of the phenotype in the cohort. This illustrates that individuals at the tail of the distribution are overrepresented among carriers of trait-increasing minor alleles and underrepresented among carriers of trait-decreasing minor alleles. The dashed, gray horizontal line indicates the overall carrier rate for both SNPs in the population (=0.51). (D) The average standard errors of allelic effects for trait-increasing and trait-decreasing minor alleles in the cohort. Because high-leverage individuals disproportionately carry trait-increasing minor alleles, and their residuals are relatively small, those effects are estimated with smaller standard errors. Bars show 95% CIs. (E) We simulated cohorts with varying trait skewness to illustrate the relationship of trait skewness and inferred sign bias. Each data point shows average sign biases and standard deviations across 20 iterations for each of 41 values cohort trait skewness. The population sign bias is zero by construction (yellow dotted line), yet skewed sampling induces a true cohort sign bias (measured by differencing the number of minor alleles with trait-increasing minor alleles and trait-decreasing minor alleles segregating in the cohort; dark purple triangles) and an even larger estimated sign bias (black points). Error bars show 95% CIs.

We illustrate this concept using a simulation study (Methods). In short, we simulated a population with an equal number of trait-increasing and trait-decreasing alleles (i.e., no true population sign bias) of equal magnitude, which segregated at equal frequencies, and investigated the chain of consequences leading from trait-based sampling to sign bias. First, individuals with high trait values are enriched for trait-increasing and depleted of trait-decreasing minor alleles (Fig. 3C). Second, carriers of minor alleles carry more influence (exert more leverage) on regression slopes in GWAS, because their genotype is farther from the genotypic mean (Fig. 3B). Individuals with high trait values therefore influence regression fits disproportionately and tend to have smaller regression residuals (Fig. 3B) producing lower uncertainty for minor alleles that they carry. Given the enrichment of trait-increasing minor alleles among high trait individuals, the estimation uncertainty (as reflected, e.g., in standard errors) is lower for trait-increasing minor alleles (Fig. 3D). When allelic effects are aggregated across variants (be that via significance filtering, shrinkage weighting or even the raw difference between the numbers of SNPs with positive and negative estimated effects), the asymmetry in uncertainties causes trait-increasing alleles to contribute disproportionally, yielding an overall positive sign bias estimate. Across simulated studies with varying degrees of skewness, we confirmed that the extent of sign bias correlates with the cohort’s phenotypic skewness (Fig. 3E; Table S6). In particular, even when there is no sign bias in the population by construction (yellow line in Fig. 3E), higher trait value-skewed sampling drives a somewhat higher true sign bias in the cohort (dark purple triangles and line in Fig. 3E) and a much higher estimated sign bias (black points and line in Fig. 3E) as a result of the asymmetry in uncertainty illustrated in Fig. A-D.

We investigated whether this prediction bears out in biobank data. Because the three biobank samples are from different countries and the study designs have somewhat different goals and recruitment approaches, the skewness varied substantially for some traits. For example, the number of schizophrenia cases varied (translating to a variable skew) across biobanks. Monocyte percentage, a quantitative trait, was markedly more skewed in UKB (7.77)*thaninAoU* (1.52). These differences likely reflect, in part, differences among biobanks in both recruitment and measurement (Discussion).

**Figure 4:**
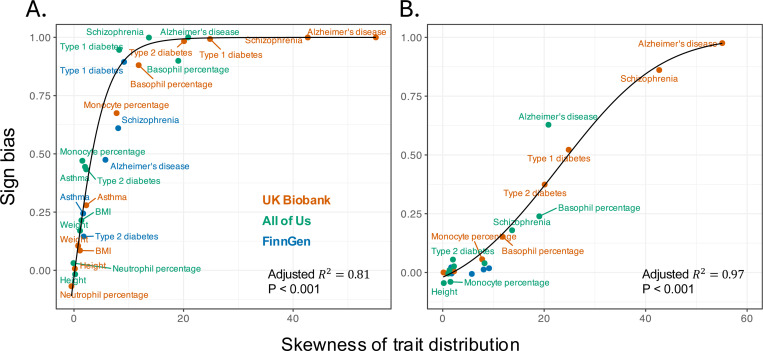
The skewness of trait distribution among study participants predicts sign bias estimates. Across traits and biobanks, the skewness (standardized third central moment) of the trait distribution predicts sign bias. Each point corresponds to a trait in one biobank. The sign bias here, as in Fig. 2B, was estimated across SNPs of minor allele frequency up to 0.1%. Morphometric traits clustered near zero skewness and zero sign bias, whereas imbalanced phenotypes (Alzheimer’s disease, schizophrenia, type 1 diabetes) exhibited high skewness and near-unity sign bias. A pooled quadratic logit fit (black curve, with respective fit statistics shown on the bottom right corner), ignoring biobank and trait identity, suggests that trait distribution skewness largely explains the relationships. (A) using the most significantly trait-associated SNPs per approximately independent LD block; (B) using randomly selected SNPs in each approximately independent LD block.

We focused again on rare minor alleles for the most trait-associated variants (minor allele frequencies 10^−3^ in Fig. 2B). The sign bias of diseases with high imbalance (Alzheimer’s disease, schizophrenia, and type 1 diabetes) was nearly one, suggesting the vast majority of minor alleles are risk-increasing. The sign bias of traits with a more moderate skew, like type 2 diabetes and basophil percentage, was smaller but also substantial. Morphometric traits with vanishingly little skewness showed no sign bias (Fig. 4A). Across traits, sign bias and phenotypic skewness are highly correlated irrespective of the biobank (pooled Spearman’s ρ=0.967, *P* < 0.001). A quadratic logit fit supported a saturating, curved relationship of sign bias with skewness (line in Fig. 4A). Across traits and biobanks, the fit based on trait skewness alone explained 81% of the variance in sign bias. Adding fixed effects of biobanks to the model yielded only a marginal gain (incremental adjusted $R^{2}=2\%$), and further adding biobank-specific slopes improved penalized fit only slightly more (incremental adjusted $R^{2}=+2.4\%$). These results suggest that trait skewness in the cohort, rather than other biobank-specific features, largely determines the estimated sign bias. This pattern held when we used different minor allele frequency thresholds when calculating sign bias (Fig. S6; Table S7-S8).

We repeated the analysis with a randomly sampled set of variants (see Methods; Fig. 4B), and again found a strong correlation between sign bias and skewness (pooled Spearman’s ρ=0.78, *P* < 0.001). A quadratic logit fit remained tight (adjusted $R^{2}=97\%$) and allowing for biobank-specific intercepts (incremental adjusted $R^{2}\approx 0\%$) or both intercepts and slopes (incremental adjusted $R^{2}\approx +0.7\%$) did not substantially improve the fit. We observed qualitatively similar results when using a different minor allele frequency cutoff (Fig. S7; Table S8).

## Discussion

Our results suggest a link between the characteristics of genetic studies and inferences about genetic architecture. We studied sign bias as a case study of this link, and in this case large differences across biobanks were remarkably well explained by a simple summary of the distribution of the trait among study participants—namely, its skewness. This finding serves as a cautionary tale: summaries of genetic architecture can be influenced by study recruitment and do not necessarily reflect trait-level properties.

Differences in representation across studies stem in large part from differences in recruitment strategies, such as how participants are enrolled into the study and how phenotypes are measured and included (e.g., population volunteers, patient-based enrollment, and disease-enriched designs^27–29^). For example, participants recruited from clinical settings often have higher representation of rare diseases, resulting in more cases in disease cohorts^58^.

Consistent with previous work^36^, we observed systematically lower SNP heritabilities in AoU compared to UKB. Chen et al.^16^ reported lower SNP heritabilities in other disease-enriched biobanks (e.g., BBJ) relative to population-based, volunteer cohorts such as UK Biobank and the Taiwan Biobank. To our knowledge, our results offer the first demonstration that the pattern of lower SNP heritability in disease-enriched biobanks holds even in ancestry-matched subsamples. One implication is that disease-enriched studies may be less powered to detect some genotype-phenotype associations compared with population-based cohorts.

What drives the systematic difference in heritability? One possibility is a systematic biological difference. For example, larger variance in effects not captured by common SNP variation (including environmental effects) among AoU participants compared with UKB participants. Another possibility is larger non-biological error in AoU, for example because of heterogeneity in trait definitions (e.g., disease classification and diagnostic codes) or measurement error. Many traits in AoU (and other disease enriched studies) are derived from electronic health records and heterogeneous clinical laboratory data. These measurements are likely less standardized across study sites and time. This hypothesis is especially plausible for biomarkers and other clinical measurements, where differences in units or reporting conventions can add noise.

Although participation has been shown to affect estimates of genetic correlations^23^, the low correlations estimated for some traits (e.g., basophil percentage and type 1 diabetes) across biobanks are perhaps surprising, all the more so given our ancestry matching. These observations help contextualize comparisons of genetic architectures and polygenic score prediction accuracy^59,60^ across ancestry groupings. Some studies attribute discrepancies solely to differences in linkage disequilibrium (LD) patterns, causal allele frequencies or allelic turnover, or heterogeneity in the causal allelic effect distributions^61–63^. When cohort recruitment and other biobank characteristics are confounded with ancestry groupings, however, as they typically are, it becomes difficult to interpret differences in allelic effects (probed, e.g., via genetic correlation), heritabilities or the predictive utility of polygenic scores^64,65^. Furthermore, since recruitment and participation can differ by genetic ancestry groups within a biobank, such factors may be confounded with ancestry in single-biobank analysis as well^64,66,67^.

Sign bias could also be affected by biological and evolutionary mechanisms beyond recruitment effects, such as biases in the sign of de-novo mutations and natural selection. For example, quantitative phenotypes can be skewed, even under neutrality, if mutational effects are asymmetric or heavy-tailed^68^. Additionally, a relationship between positive sign bias and allele frequency might be expected for traits (especially diseases) under purifying selection, such that risk-increasing mutations are constrained to lower frequencies^1,12,13,49,51,69^. This expectation matches our observations of mean sign bias across the minor allele frequency spectrum, which show stronger signals for disease phenotypes. However, such mechanisms should be largely cohort-invariant and they do not explain why the same trait’s sign bias would differ across GWAS with similar sample composition of genetic ancestry. For a selective explanation to hold, selection would have to differ dramatically across countries and produce detectable changes to genetic architecture over contemporary timescales and tiny population divergences^70–76^, which seems implausible. By contrast, our data show that cross-cohort differences in sign bias correlate with cohort-specific phenotypic skewness (which is considerably higher for binary disease traits), rather than biobank identity per se. This relationship would be expected from a study design mechanism, in that properties of the trait distribution influences allelic effect estimation, thereby modulating the observed sign bias on top of any evolutionary baseline.

Throughout, we analyzed allelic effects using raw trait units of continuous traits rather than transforming the phenotypes. In contrast, many GWAS analyses transform quantitative trait data (typically via inverse-rank normalization) so that the residuals are closer to conditionally Gaussian and measurement units are standardized across traits^77^. Such a transformation is useful for enhancing discovery power and producing “well-behaved” test statistics^78^. For example, inverse-rank normalization of the trait distribution would remove trait skewness and, as we show (Fig. S8), result in little-to-no sign bias. It is therefore unsurprising that, to our knowledge, most studies that have not found pronounced sign bias in allelic effects from GWAS for highly symmetric phenotypes, transformed or not (see for example the symmetric ”smile” plot of effect size vs. allele frequency for trait-increasing alleles reported by Koch et al^51^). However, transforming data (rather than using the scale deemed biologically relevant) can render allelic effect estimation and inferences about genetic architectures more mathematically convenient but less biologically interpretable. In addition, we note that binary traits such as disease status are not typically transformed, and the interpretation of summaries such as sign bias on a linear liability scale may remain impacted by participation in genetic studies.

In this work, we challenge an often implicit assumption that inferences about the genetic architecture of traits and diseases are not biased by representation in genetic studies. We argue instead that such inferences are best viewed as cohort-dependent summaries rather than trait-intrinsic properties. Similarly, nominally “cross-ancestry” or “trans-ethnic” comparisons should be interpreted with caution, as apparent differences may partly reflect variation in recruitment or participation. Going forward, it will be important to evaluate what facets of genetic architecture replicate across studies and why. It therefore seems vital to broaden the research community’s access to studies with diverse recruitment and participation profiles. This would help ensure that inferences about genotype-complex phenotype maps are generalizable and ultimately benefit everyone.

## Methods

### Data.

We assembled a panel of traits that are present in both the UK Biobank (UKB) and All of Us (AoU) databases. The UKB cohort comprises approximately 500,000 residents of the United Kingdom aged 40–69 recruited between 2006–2010^27^. The AoU program is a nation-wide, longitudinal cohort in the United States aimed at recruiting participants from a diversity of ancestral backgrounds^28^. The database currently contains over 800,000 participants over 18 years of age, over 400,000 of which currently have whole-genome sequences available. We chose to analyze 11 traits including three common morphometric traits (height, weight, and BMI), three immunological traits (monocyte percentage, basophil percentage, and neutrophil percentage), and five common diseases (asthma, type 1 diabetes, type 2 diabetes, schizophrenia, and Alzheimer’s disease).

For AoU, we used traits under the version 8 release in the Curated Data Repository (CDR). For physical measurements (height and weight), we queried the AoU CDR measurement table for records with ”measurement_source_value” equal to “height” or “weight”. We restricted to rows with interpretable units by retaining measurements recorded in centimeters (height) and kilograms (weight) and excluded records with missing values or non-matching/unknown units. We then computed BMI per participant as weight (*kg*) divided by height ($m^{2}$). For white blood cell traits (monocyte, basophil, and neutrophil percentages), we queried the measurement table using LOINC codes 5905–5 (monocytes), 706–2 (basophils), and 770–8 (neutrophils). We kept only records whose unit was a percentage (unit concept name in “percent”, “percentage unit”, “percent of white blood cells”) to match UKB definitions (calculated as the proportion of the given biomarker in the leukocytes; UKB data fields 30190 for monocyte percentage, 30220 for basophil percentage, and 30200 for neutrophil percentage). We further excluded records with missing values and excluded measurements with values lower than 0 or higher than 100. Because participants can have repeated laboratory measurements, we summarized each participant’s phenotype as the median of available values across time. For diseases in AoU, we pulled disease status from electronic health record (EHR) data by mapping ICD codes to phecodes using Phecode map X (https://phewascatalog.org/), requiring at least two distinct dates with a mapped code for case assignment.

To broaden the scope of the analysis, we additionally included matched disease endpoints from FinnGen, a nationwide Finnish research collaboration that connects genomic data (from genotype arrays and imputation) to decades of digital health registries in Finland for over 500,000 participants^29^. Since we did not have access to individual-level FinnGen data, we only used it in the analysis of sign bias presented in Figs. 2, 4 and further only used disease traits for which we can calculate the skewness based on reported incidence, without individual level data. More details on the traits used are in Table S1.

### GWAS.

For UKB, we downloaded the GWAS summary statistics from the Neale Lab (http://www.nealelab.is/uk-biobank/). These consisted of 13.7 million SNPs both including genotype array and imputed SNPs which are based on a subsample consisting of 361,194 unrelated individuals who cluster together in genetic principal component space (within a 7 standard deviation radius of individuals of “white British” ancestry) and who self-reported as white and British, Irish, or white ethnicity. For quantitative traits, we used summary statistics for the raw, untransformed phenotypes. We additionally used the public release of FinnGen GWAS summary statistics (DF12 release), performed on 21,311,644 array and imputed SNPs using 500,348 samples for the 5 disease endpoints (https://r12.finngen.fi/). For all downstream analyses for UKB and FinnGen, we removed non-biallelic variants, variants with INFO scores < 0.8, variants with a Hardy Weinberg exact test *P* < 1 × 10^−10^, and variants with a minor allele count ≤ 20.

For AoU, we performed a GWAS ourselves on the same 11 traits, but sought to first identify a sample with similar ancestry composition to that used to generate the Neale Lab’s UKB-based summary statistics. We projected both the UKB and AoU individuals into the same PC space using pre-computed SNP loadings provided by the Global Biobank Meta-analysis Initiative^79^. We removed individuals who were flagged for QC issues by AoU. We removed close relatives (estimated kinship coefficient of more than 0.1, which covers first and second degree relatives) using summaries provided by AoU. We excluded individuals whose self-reported gender was not man or woman or whose self-reported sex was not male or female. We then trained various classifiers on the UKB cohort to predict white British-like ancestry using the first 10 PCs from the shared PC space. The random forest classifier (F1-score 0.97) was used to identify the ancestry-matched cohort from AoU (*n* = 221, 105). Self-reported race was used to validate that results indicate majority-similar self-reported race categories between the UKB and AoU samples (in this case ‘White-British’ and ‘White’), but were not used to determine inclusion in the matched AoU sample. Additionally, for each phenotype in our analysis, we removed individuals with missing values for that phenotype. We were left with between 69,249 to 199,529 AoU individuals for each phenotype.

We performed all GWAS using linear regression with *PLINK* 2.0^80^ on the raw phenotypic values for each trait. We included the covariates age, age^2^, sex, sex×age, sex×age^2^, and the first 16 principal components of the genotype matrix of the ancestry-matched sample (all covariates were standardized to have mean 0 and variance 1 by using the *PLINK* flag –covar-variance-standardize). We used SNPs from the All of Us Allele Count/Allele Frequency (ACAF) subset of the whole-genome sequence data. We performed the analysis on a set of 10,102,149 matched variants (based on dbSNP rsids) in both the UKB and AoU datasets. We excluded variants with a call rate < 95%, and excess-heterozygosity outliers using a one-sided mid-p Hardy–Weinberg exact test (flag keep-fewhet) with threshold *P* < 1 × 10^−12^. For all downstream analyses we only used summary statistics for bi-allelic SNPs and only kept variants with minor allele counts > 20 in the final ancestry-matched sample.

### Estimates of genetic architecture summaries.

Using GWAS summary statistics, we estimated narrow-sense heritability using an additive SNP model for each trait and the genetic correlations between biobanks using LD Score Regression^30,43^. We calculated liability scale estimates of SNP heritability for binary traits, assuming that the population prevalence matches the prevalence in the given biobank. We estimated effective polygenicity for each trait with S-LD4M^31^. For all analyses, we used pre-calculated LD scores for HapMap3 variants and for the European ancestry group as provided by Bulik-Sullivan et al^30^.

### Estimating sign bias.

For each trait, we polarized GWAS-estimated SNP allelic effect such that the sign represents that of the minor allele relative to the major allele. We estimated posterior sign probabilities for each SNP included in the GWAS using adaptive shrinkage (*ash*^54^). We estimate the posterior sign of the allelic effect for variant i as

(1) $$\eta_{i}=Pr\left(\beta_{i}>0|\hat{\beta },s,\hat{\pi }\right)-Pr\left(\beta_{i}<0|\hat{\beta },s,\hat{\pi }\right),$$

where $Pr\left(\beta_{i}>0|\hat{\beta },s,\hat{\pi }\right)$ is the posterior probability (estimated using *ash*) that the minor allele of variant i is trait-increasing, and $Pr\left(\beta_{i}<0|\hat{\beta },s,\hat{\pi }\right)$ is the posterior probability that it is trait-decreasing. In both terms, $\beta_{i}$ denotes the (unknown) true effect for SNP i, $\hat{\beta }$ is the vector of observed minor-allele effects estimated from all GWAS SNPs, s the estimated standard errors of $\hat{\beta }$, and $\hat{\pi }$ denotes the empirical Bayes mixture weights over a set of zero-centered Normal distributions with distinct variances. $\eta_{i}$ approaches +1 (−1) with increasing certainty that the minor allele is trait-increasing (decreasing) and nears 0 either when there is high uncertainty about the sign or the posterior probability that there is no effect is high.

The estimation of the sign bias in a given set of SNPs can be confounded by linkage disequilibrium (LD) among SNPs in a set. To avoid this confounding, we only use one SNP per approximately independent LD block following the protocol used in Zhu et al^81^. For the UKB, we used the 1,703 blocks for the European subset (EUR ”superpopulation”) of the 1000 Genomes Phase 3 dataset^72^ as inferred by Berisa and Pickrell^82^. For AoU, we used the updated map of 1,361 approximately independent LD blocks on the EUR individuals for build hg38 from MacDonald et al^83^.

For a set of SNPs k, we select a single variant with the smallest association *p*-value from each of b LD blocks and average SNP sign estimates with weights proportional to their certainty as

(2) $${\hat{\eta }}_{k}=\frac{\sum_{i=1}^{b}\eta_{i}}{\sum_{i=1}^{b}\left|\eta_{i}\right|}.$$

${\hat{\eta }}_{k}$ approaches +1 when all the minor alleles in set k are trait-increasing, −1 when they are all trait-decreasing, and 0 when evidence is equal for trait-increasing and trait-decreasing effects. Within each bin, we only retained LD blocks that contained at least 10 SNPs. We obtained bootstrapped standard errors for ${\hat{\eta }}_{k}$ by resampling the b contributing LD blocks with replacement, selecting the SNP with the lowest *p*-value within each resampled block, and recomputing Eq. 2 1,000 times. For each set k, we used the mean value of ${\hat{\eta }}_{k}$ as the point estimate for the sign bias and the bootstrap sample standard deviation (across 1,000 estimates) as its standard error.

### Simulating traits and associated allelic effects.

To understand how trait skewness can drive sign bias, we simulated genotypes and phenotypes in a population, and performed GWAS in samples chosen with varying trait skewness and examined downstream GWAS statistics. This allowed us to isolate a mechanism by which the trait distribution skewness can generate sign bias, even when there is no sign bias in the generative process.

We simulated a population ($N=2\times 10^{6}$ individuals) with equal numbers of independent trait-increasing and trait-decreasing alleles with equal magnitudes and frequencies, consisting of M=2,000 total variants. For each pair, we drew a minor allele frequency p log-uniformly between $5\times 10^{-4}$ and $8\times 10^{-3}$ and assigned this same frequency to both variants in the pair. We assigned each variant j an effect of $\beta_{j}$, where $\left|\beta_{j}\right|=\sqrt{h^{2}/M}$ for all j and $h^{2}=0.6$, with the first 1,000 variants positive in sign and second 1,000 variants negative in sign. We drew genotypes under Hardy-Weinberg equilibrium to yield the genotype dosages, G, in individual i for variant j as

(3) $$G_{ij}∼Binomial(2,p).$$

SNPs were assumed independent of one another (no linkage disequilibrium) and individuals were simulated as unrelated (no kinship). Genotype dosages were standardized to mean 0 and variance 1.

To provide a clear contrast for visualization purposes (Fig. 3C), we added a pair of focal variants d at a minor allele frequency of $p_{d}=0.3$ with equal effect magnitudes of $\left|\beta_{d}\right|=0.4$, but with one variant assigned a negative sign and one a positive sign. The standardized dosages in individual i of the trait-increasing and trait-decreasing focal variants are thus $G_{i}^{+}$ and $G_{i}^{-}$ respectively.

Adding in individual environmental noise $\epsilon_{i}∼N\left(0,1-h^{2}\right)$, and the phenotype for individual i is defined as:

(4) $$Y_{i}=\sum_{j=1}^{M}G_{ij}\beta_{j}+\beta_{d}\left(G_{i}^{+}-G_{i}^{-}\right)+\epsilon_{i},$$

which was scaled across the population to mean 0 and variance 1.

Cohorts were drawn from this population as follows: We partitioned the population into B=200 equal-quantile bins based on the population trait value Y. Let b∈{1,…,B} index bins, and define each bin’s mid-quantile rank as

(5) $$r_{b}=\frac{b-0.5}{B}$$

We assigned each bin a weight

(6) $$w_{b}={\left(1-r_{b}\right)}^{\gamma },$$

where γ is a shape parameter that controls how strongly sampling probability depends on mid-quantile rank, such that larger values increase the right skew in the sampled cohort. We mixed this weighted distribution with a uniform component, τ, to ensure all bins were sampled. The marginal sampling probability per bin becomes

(7) $$p_{b}=\frac{\tau }{B}+(1-\tau )\frac{w_{b}}{\sum_{i=1}^{B}w_{b}}.$$

Given $p_{b}$ and a cohort sample size of 10,000, we allocated per-bin sample counts via a multinomial draw and then sampled individuals within each bin without replacement. We performed genome-wide association within each cohort using ordinary least squares regression for each variant after centering the cohort phenotype and cohort genotypes per variant. We partitioned the variants into contiguous pseudo-blocks of 6 SNPs and selected either the most significant variant per block (smallest two-sided *p*-value from calculated from variant z-scores) or a random SNP per block. Sign bias was computed using Eq. 1 on each selection via output of adaptive shrinkage (*ash*) as described in the section Estimating sign bias. We used Eq. 2 to estimate the certainty-weighted sign bias for the cohort.

We repeated cohort sampling and GWAS across a predefined grid of target phenotype skewness values ranging from 0 to 4 in increments of 0.1. To ensure these varying target skewness values could be reached, we optionally allowed for a threshold parameter q, retaining only bins with $r_{b}\geq q$, and we performed a grid search over these sampling parameters (q,γ) with τ=0.1 to identify values that produced cohort skewness closest to the target values. For each skewness value, we generated 20 independent cohorts by resampling individuals from the population. Because the achieved skewness of each sampled cohort differed slightly from its target, realized skewness values were binned, and within-bin means and standard errors of sign bias were computed.

### Evaluating the relationship between trait skewness with sign bias.

We processed traits in the UKB to correspond to their GWAS equivalents by following the sample QC procedures outlined by the Neale Lab (for the UKB v3 GWAS; http://www.nealelab.is/uk-biobank/). For AoU, we used the trait distribution for individuals who passed our sample QC steps outlined above in **GWAS**. For FinnGen disease endpoints, information about the number of cases vs non-cases used in each GWAS is publicly available^29^. For each trait distribution, we calculated the standardized third moment (skewness). Importantly, we did not apply any normalization or covariate residualization: skewness is evaluated on the observed scale within each biobank.

We paired the computed phenotypic skewness of the raw phenotypes with sign bias estimates for alleles with frequencies ≤ 10^−3^ (these estimates are shown in Fig. 2B and Fig. S4 calculated from Eq. 2). In the notation below, we designate this sign bias estimate for a given trait T from biobank g as ${\hat{\eta }}_{T,g}$. Because ${\hat{\eta }}_{T,g}$ lies between −1 and 1, and our observed estimates lie between approximately 0 and 1 (see Fig. 4), we map ${\hat{\eta }}_{T,g}$ to a probability scale (the probability that an allele is trait-increasing) and analyze the log-odds. We modeled the association between phenotype skewness and sign bias with a second-degree polynomial such that

(8) $$Z_{T,g}=\beta_{0}+\beta_{1}\gamma_{T,g}+\beta_{2}\gamma_{T,g}^{2}+\epsilon ,$$

where $Z_{T,g}=\mathrm{logit}\left(\frac{{\hat{\eta }}_{T,g}+1}{2}\right)$, $\gamma_{T,g}$ represents the skewness of trait T in biobank $g,\beta_{0}$ is the global intercept, $\beta_{1}$ and $\beta_{2}$ are the slopes, and ϵ are mean-zero residuals. We also fit a model that allowed for biobank-specific intercepts as

(9) $$Z_{T,g}=\beta_{0}+\sum_{g}\delta_{g}D_{g}+\beta_{1}\gamma_{T,g}+\beta_{2}\gamma_{T,g}^{2}+\epsilon ,$$

where $D_{g}$ is an indicator variable for biobank g (either UKB, AoU, or FinnGen), with UKB used as the reference, and $\delta_{g}$ is the intercept shift for biobank g. Finally, we fit a model that allowed for biobank interactions as

(10) $$Z_{T,g}=\beta_{0}+\sum_{g}\delta_{g}D_{g}+\sum_{g}\left(\beta_{1,g}\gamma_{T,g}+\beta_{2,g}\gamma_{T,g}^{2}\right)D_{g}+\epsilon ,$$

where $\beta_{1,g}$ and $\beta_{2,g}$ are the biobank-specific slopes.
